# Predicting miRNA Targets by Integrating Gene Regulatory Knowledge with Expression Profiles

**DOI:** 10.1371/journal.pone.0152860

**Published:** 2016-04-11

**Authors:** Weijia Zhang, Thuc Duy Le, Lin Liu, Zhi-Hua Zhou, Jiuyong Li

**Affiliations:** 1 School of Information Technology and Mathematical Sciences, University of South Australia, Adelaide, South Australia, Australia; 2 National Key Labotorary, Nanjing University, Nanjing, Jiangsu, China; Kunming University of Science and Technology, CHINA

## Abstract

**Motivation:**

microRNAs (miRNAs) play crucial roles in post-transcriptional gene regulation of both plants and mammals, and dysfunctions of miRNAs are often associated with tumorigenesis and development through the effects on their target messenger RNAs (mRNAs). Identifying miRNA functions is critical for understanding cancer mechanisms and determining the efficacy of drugs. Computational methods analyzing high-throughput data offer great assistance in understanding the diverse and complex relationships between miRNAs and mRNAs. However, most of the existing methods do not fully utilise the available knowledge in biology to reduce the uncertainty in the modeling process. Therefore it is desirable to develop a method that can seamlessly integrate existing biological knowledge and high-throughput data into the process of discovering miRNA regulation mechanisms.

**Results:**

In this article we present an integrative framework, CIDER (**C**ausal m**i**RNA target **D**iscovery with **E**xpression profile and **R**egulatory knowledge), to predict miRNA targets. CIDER is able to utilise a variety of gene regulation knowledge, including transcriptional and post-transcriptional knowledge, and to exploit gene expression data for the discovery of miRNA-mRNA regulatory relationships. The benefits of our framework is demonstrated by both simulation study and the analysis of the epithelial-to-mesenchymal transition (EMT) and the breast cancer (BRCA) datasets. Our results reveal that even a limited amount of either Transcription Factor (TF)-miRNA or miRNA-mRNA regulatory knowledge improves the performance of miRNA target prediction, and the combination of the two types of knowledge enhances the improvement further. Another useful property of the framework is that its performance increases monotonically with the increase of regulatory knowledge.

## Introduction

miRNAs are short non-protein coding RNAs that regulate gene expression by either marking their target mRNAs for degradation or repressing translation. miRNAs mainly identify their target mRNAs by binding to the 3’-untranslated region (3’ UTR) or 5’ UTR. Studies have shown that miRNAs play important roles in a broad range of biological processes, such as differentiation [1], development [2], apoptosis [3] and cellular signaling [4]. Because of their biological importance, miRNAs are related to a variety of diseases, such as cancer and cardiovascular diseases [5]. Therefore, precise identification of miRNA targets is critical to the understanding of the functions of miRNAs in both healthy and diseased tissues [6, 7].

Computational approaches are a necessary and promising way to help unveil the complete picture of miRNA regulatory relationships. Significant progress has been made in elucidating the relationships between miRNAs and their targets using wet-lab biological experiments [8–11]. However, it is unrealistic to hope for a complete picture of miRNA regulation mechanisms by relying solely on wet-lab experiments due to the huge number of possible relationships and high expenses of the experiments [12]. Therefore, dry-lab approaches have been considered as a cost-effective and promising alternative and have shown great promise in identifying putative miRNA targets [13–16].

Because of the large number of miRNAs and mRNAs involved in gene regulation, providing reliable predictions has always been a significant challenge for computational biology approaches. This problem is further exacerbated by the small number of available samples. Therefore researchers have to rely on the integration of biological knowledge and data driven discovery process to obtain a complete understanding of miRNA regulation mechanisms.

Bayesian network (BN) [17–22] provides an excellent platform for seamless integration of prior knowledge and data in the process of causal structure learning. Furthermore, the causal semantics of a BN makes it a preferred model for representing gene regulatory networks since the interactions among genes are causal relationships rather than statistical associations.

Valuable wet-lab validated knowledge cannot be effectively utilised with the existing methods [23–26]. These algorithms use prior knowledge to restrict their search space in the way that the knowledge is used to initialise the structure of a BN and the learning process is aimed at removing false positives from the initial structure [27–31]. Therefore the final structure is a sub-graph of the initial one and a miRNA-mRNA interaction will not be predicted if it is not included in the prior knowledge. Consequently such methods usually require users to have a large amount of knowledge which covers the complete or nearly complete knowledge of the network structure, and are not able to utilise the sparse and limited validated knowledge.

In this paper, we propose the CIDER framework to effectively utilise sparse wet-lab validated knowledge, including transcriptional miRNA-mRNA and post-transcriptional TF-miRNA regulatory knowledge [32]. Our method differentiates from the existing work in two aspects: first instead of using the regulatory knowledge to initiate the network structure and then remove false positive edges, we enforce the learning process to maintain the experimentally confirmed relationships without restricting the search space. Secondly the regulatory knowledge is used for the purpose of obtaining more accurate estimation of the causal effect of miRNAs on mRNAs, whereas existing methods use prior knowledge to learn the causal regulatory structure.

Our results on both real-world and simulated datasets demonstrate that a very small amount of validated regulatory knowledge improves the accuracy of predicted miRNA targets significantly, and the performance of CIDER increases monotonically with the increase of regulatory knowledge.

We show that when wet-lab validated knowledge is analysed together with expression profiles, CIDER discovers significantly more validated miRNA targets than using expression profiles alone. It is also shown that either TF-miRNA or miRNA-mRNA regulatory knowledge improves the performance, and the combination of the two types of knowledge enhances the performance further.

**Fig 1:**
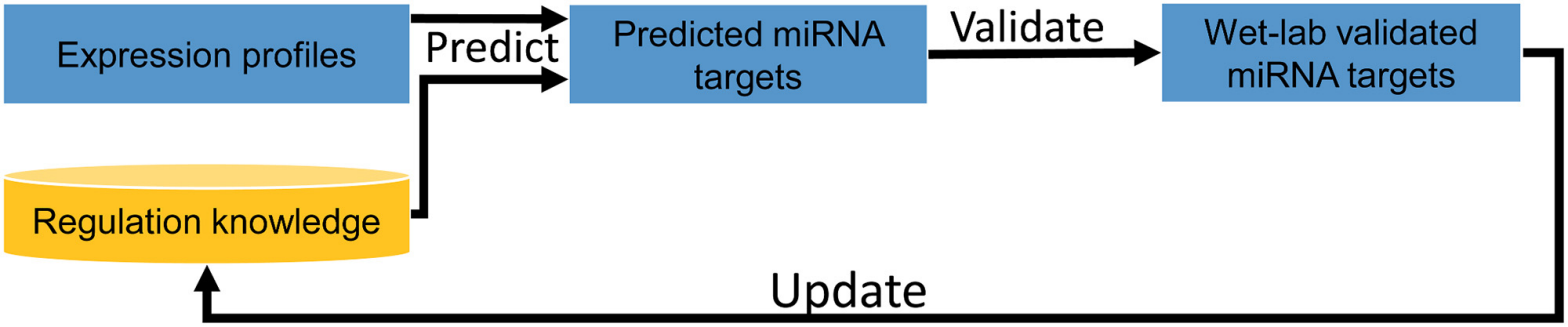
An iterative process of integrating and discovering miRNA regulatory relationships. Our proposed framework is one iteration of the above knowledge and data integrated discovery process. In the long run, wet-lab and dry-lab discoveries become an integrated feedback process for uncovering new biological insights. Bayesian network based causal reasoning provides an excellent platform for a seamless integration.

An important property of the framework is that the performance of miRNA target prediction improves monotonically with the amount of regulatory knowledge used. In other words CIDER makes more reliable discoveries from the data when the knowledge integrated into the framework increases. In Fig 1, we illustrate a promising knowledge discovery process based on this property. With the incorporation of regulatory knowledge in CIDER, the process becomes a feedback loop for the discovery of new biological hypotheses and it naturally combines dry-lab predictions with web-lab experiments.

## Materials

### Matched expression profiles

#### NCI-60 data for Epithelial to Mesenchymal Transition (EMT)

The EMT [33] dataset includes the miRNA expression profiles for the NCI-60 panel cell lines from [34], and the dataset is available at http://www.ncbi.nlm.nih.gov/geo/query/acc.cgi?acc=GSE26375. The mRNA expression profiles for NCI-60 were downloaded from ArrayExpress available at http://www.ebi.ac.uk/arrayexpress, accession number E-GEOD-5720. We use the cell lines categorized as epithelial (11 samples) and mesenchymal (36 samples) in this study.

#### Data of the 51 human breast cancer cell lines (BRCA)

The BRCA dataset includes miRNA expression profiles from the breast cancer cell lines data provided by [35]. The mRNA expression profiles for these cell lines can be downloaded from http://www.ncbi.nlm.nih.gov/geo/query/acc.cgi?acc=GSE41313. 27 samples in the luminal group and 23 samples in the basal group are used.

### Gene regulation databases

#### TF-miRNA interaction database

For transcriptional regulatory knowledge, we use TransmiR [36], a TF-miRNA regulatory relationships database including approximately 700 entries manually collected from relevant literatures. This database is available online at http://www.cuilab.cn/transmir.

#### Experimentally validated miRNA-mRNA interaction databases

The post-transcriptional regulatory knowledge is obtained from miRNA target databases Tarbase v6.0 [37], miRTarbase v4.5 [13] and miRWalk [38]. Tarbase and miRTarbase contain experimentally confirmed miRNA target information manually collected from related literatures. miRWalk contains both predicted and validated miRNA targets, but we only utilise the experimentally validated targets in our experiments. The detailed information of experimentally validated miRNA-mRNA interactions retrieved from all these databases can be found in S3 File.

#### Predicted miRNA-mRNA interaction database

We also utilise TargetScan v6.2 [39], a commonly used miRNA target prediction database. TargetScan predicts miRNA targets by searching for binding sites that match the seed region of each miRNA. This database is available online at http://www.targetscan.org.

## Methods

### Notation

Let G=(V,E) denote a *graph* where **V** = {*X*_1_, …, *X*_*l*_} is a set of *vertices* and **E** ⊆ **V** × **V** is a set of *edges*. In our framework, the vertex set **V** represents a set of random variables corresponding to the expression levels of miRNAs and mRNAs (including TF coding mRNAs), and the edges represent the causal relationships between the variables.

We use *X*_*i*_ → *X*_*j*_ or *X*_*i*_ ← *X*_*j*_ to represent a directed edge between *X*_*i*_ and *X*_*j*_. *X*_*i*_ − *X*_*j*_ is used to represent an undirected edge between *X*_*i*_ and *X*_*j*_. The set of all parent nodes of *X*_*j*_ is denoted as *pa*_*j*_. A *directed* graph is a graph in which all edges are directed. An *undirected* graph is a graph in which all edges are undirected. We say that a graph G is *acyclic* if and only if all its directed edges do not form any cycle in G. In this article, we always assume the graph is acyclic.

### The proposed CIDER framework

**Fig 2:**
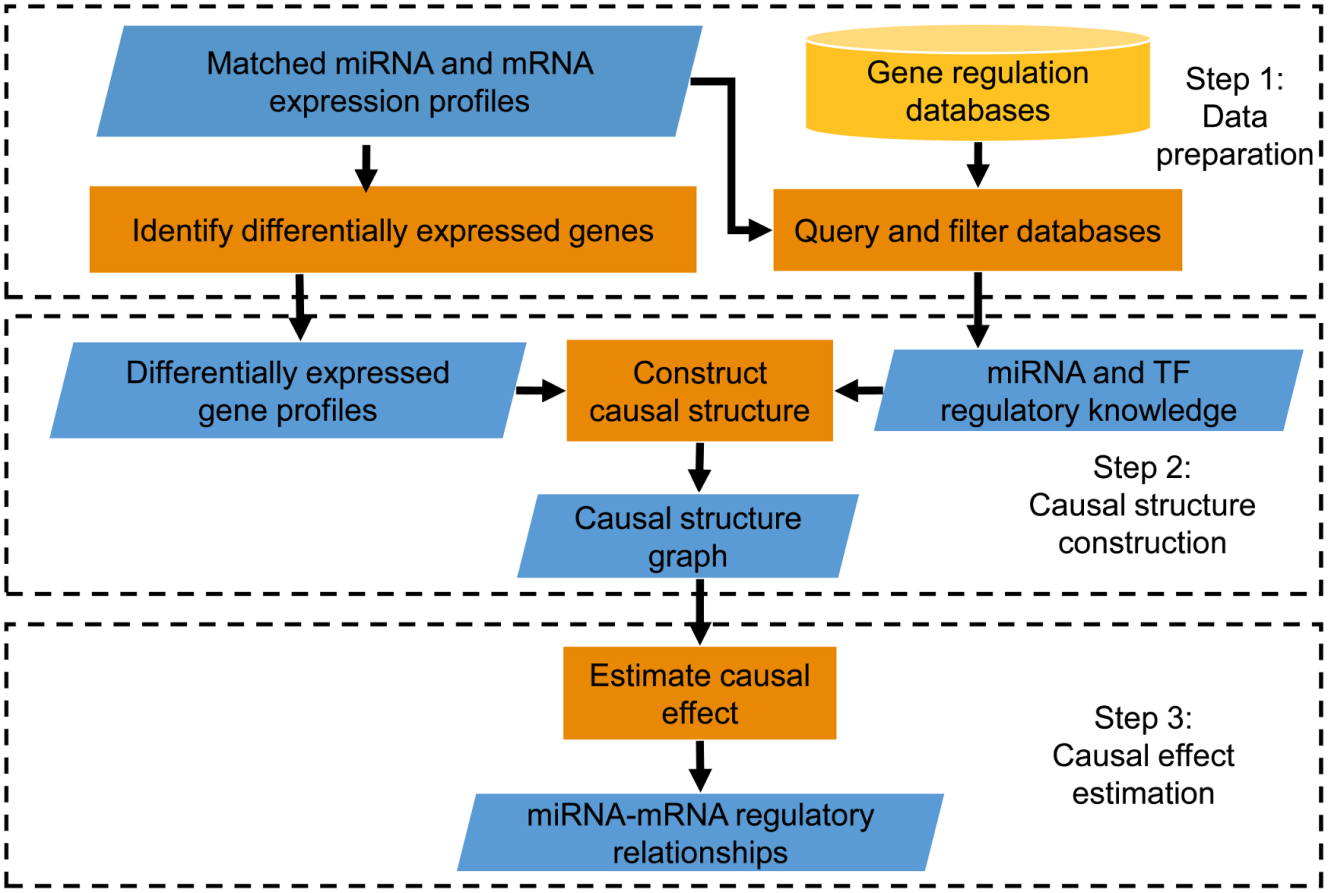
The proposed CIDER framework. First the differentially expressed miRNAs and mRNAs are selected in the expression profiles [40], then we query the regulatory databases for gene regulation knowledge. After that we build the causal structure according to the expression profiles and the knowledge, followed by the causal inference to identify miRNA-mRNA interaction pairs.

As illustrated in Fig 2, the CIDER framework consists of three steps. In the first step we perform differential gene expression analysis and query the databases for gene regulation knowledge. To identify the targets of a miRNA, we use *do-calculus* [18] to estimate the causal effects the miRNA have on all the mRNAs. In other words, *do-calculus* estimates how the expression values of the mRNAs change when the expression of the miRNA is intervened [41]. In order to apply *do-calculus*, we need to know the causal relationships between the variables. Therefore in Step 2 we construct the causal structure with the incorporation of regulatory knowledge, then we identify the miRNA targets using *do-calculus* in Step 3.

#### Step 1 (Data preparation)

The differential expression analysis is performed as described in [42]. As a result for the EMT dataset, 35 miRNA probes and 1154 probes of mRNAs are identified as significantly differentially expressed. For the BRCA dataset, 92 miRNA probes and 1500 mRNA probes are identified. The detailed result can be found in S1 File.

After differential expression analysis, we extract the regulatory knowledge (i.e. TF-miRNA and miRNA-mRNA interactions) relevant to the differentially expressed expression profiles from the regulatory knowledge databases described previously.

#### Step 2 (Casual structure construction)

Using both the gene regulation knowledge and gene expression data, we learn a causal Bayesian network (CBN) which models the structure of the gene regulatory network. A CBN consists of a pair <G,P>, where G is a directed acyclic graph with the differentially expressed miRNAs and mRNAs as its vertices, and *P* is the joint probability function of the vertices. An edge in G indicates a causal relationship between the two vertices. For example, an edge directing from a miRNA to a mRNA means that the miRNA regulates the mRNA; and an edge directing from a TF coding mRNA to a miRNA indicates the TF regulates the miRNA.

A common way to learn the causal structure is to start from a completed graph, then update the graph according to the gene expression data. In order to integrate the regulatory knowledge, in CIDER we label all the edges given in the regulatory knowledge as *constant edges*, which are never to be removed or altered (in terms of their directions) during the entire structure construction step.

#### Step 3 (Causal effect estimation)

We estimate the causal effect that each miRNA has on all the mRNAs according to the causal structure and expression profiles. The causal effects measures when the expression level of a certain miRNA changes, how the expression level of other mRNA will change. For each miRNA, we choose the mRNAs with the largest causal effects as the predicted targets.

In the rest of this section, we discuss the details and intuitions of Step 2 and Step 3.

### Causal structure construction

There are two steps involved in constructing a causal structure: determining the existence of edges between the nodes, and orienting the direction of the edges.

A common way [43, 44] to determine whether an edge exists between two nodes is conditional independence (CI) tests. More specifically, starting from a fully connected graph, we use CI tests to determine the dependency between all connected nodes pairs. If two nodes become independent when conditioned on any subsets of their neighbours, the edge between them is removed from the graph. Otherwise, the edge will remain in the causal structure.

During this procedure, edges may be incorrectly removed or maintained. Because the number of available samples is limited when comparing to the large number of variables in expression profiles, CI tests may declare two nodes are independent even if a dependency exists, thus the edge between them will be removed correctly. Furthermore, the incorrectly removed edges will not appear in the conditioning sets of later CI tests, which may lead to false positives (i.e. two nodes would have been tested to be independent and their edge would have been removed if the incorrectly removed edges were kept and were in the conditioning set of the CI test).

In order to determine the orientation of edges we need to identify the *v*-structures in the causal structure defined as follows:

**Definition 1 ([18])** *A triple (X_i_, X_j_, X_k_) forms a v-structure in graph* G *if and only if it suffices both of the following conditions*:

1. *X_i_ and X_j_ as well as X_j_ and X_k_ are adjacent, X_i_ and X_k_ are not adjacent*,
2. *X_i_ and X_k_ are not independent when conditioned on X_j_*.

When a *v*-structure (*X*_*i*_, *X*_*j*_, *X*_*k*_) is identified, the edges can then be oriented as *X*_*i*_ → *X*_*j*_ ← *X*_*k*_ [18]. After all the *v*-structures have been identified and oriented, we can orient the remaining edges according to the principle of avoiding the creation of cycles and new *v*-structures [44].

Unfortunately, under most circumstances the above strategy can only orient some of the edges, leaving many undirected. Undirected edges introduce uncertainty in the next step, since the estimation has to be done on all possible orientations of the undirected edges and take the lower bounds as the inferred causal effects [31].

In our framework, we utilise regulatory knowledge to alleviate both the false edges and the undirected edges problems. We introduce the concept of *constant edge*. A *constant edge* is an edge between two nodes where their relationship are already validated via biological experiments, so the edge will never be removed no matter what result the CI tests are, and the direction of the edge can be correctly determined according to the knowledge. Now let us have a look at benefits of introducing constant edges with the following example.

**Fig 3:**
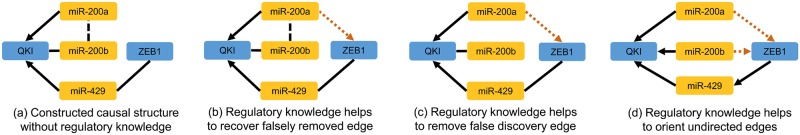
An illustration of how the prior knowledge helping the causal structure construction. Solid/dashed black lines indicate the edges correctly/incorrectly detected during the causal structure construction without the prior knowledge; Dotted brown lines indicate the edges added based on prior knowledge.

With the introduction of constant edges, we are able to recover incorrectly removed edges and also remove some falsely discovered edges. Fig 3A shows a causal structure learned with CI tests only, which includes one falsely identified regulatory relationship (miR-200a to miR-200b) and two missed regulatory relationships (miR-200a to ZEB1 and miR-200b to ZEB1). Since it is has been experimentally confirmed that ZEB1 is a target of miR-200a, we mark the edge from miR-200a to ZEB1 as a constant edge and do not remove it when using CI tests (see Fig 3B). Because of the introduction of the edge from miR-200a to ZEB1, the falsely discovered edge from miR-200a to miR-200b is removed (see Fig 3C) as the result of the conditional independence test with ZEB1 being added to the conditioning set.

Constant edges can also help to orient more undirected edges. For example, in Fig 3C, although we have removed the false edge between miR-200a and miR-200b, the directions of the two edges (miR-200b/QKI and miR-429/ZEB1) still cannot be determined. However, when we have another constant edge that miR-200b regulates ZEB1 from the regulatory knowledge, we can orient the two edges as in Fig 3D otherwise a new v-structure (at ZEB1) or a cycle (miR-200b → ZEB1 → miR429 → QKI → miR-200b) will be introduced, either of which is not allowed acyclic assumption [43].

As shown above, even when only one or two constant edge is introduced, the uncertainness in the causal structure can be significantly reduced. We briefly summarise the procedure of constructing the causal structure in Algorithm 1 (The details of the algorithm can be found in S6 File).

**Algorithm 1** Construct the causal structure G

**Input**: Gene expression profile, regulatory knowledge matrix.

**Output**: Constructed causal structure G

 Initiate G as a fully connected graph

 **//Mark constant edges**

 Mark all constant edges in G according to the regulatory knowledge matrix.

 **//Removes edges from G using CI tests**

 Test conditional dependence among non-constant edges, remove an edge between two vertices if they are found independent.

 **//Orient constant edges**

 Orient constant edges according to regulatroy knowledge

 **//Orient remaining edges**

 Identify and orient all *v*-structures

 Orient remaining edges without creating new v-structure and cycle

**return** G

### Causal Effect Estimation

With the expression data and the causal structure among its variables, we need to infer the causal effects that a miRNA has on a mRNA. By assuming all variables in the expression profiles follow the multivariate Gaussian distribution, we can calculate the causal effects as follows:

**Theorem 1 ([45])** *Let X_1_, …, X_p_, X_p + 1_, …, X_p + q_ be jointly normal distributed. The causal effect of X_i_(i = 1, …, p) on X_j_(j = p + 1, …, p + q), ce(X_i_, X_j_) can be calculated as*:

$$\begin{array}{c} ce\left(X_{i},X_{j}\right)=\beta_{ij|pa_{j}}=\left\{\begin{array}{cc} 0 & X_{j}\in pa_{i}\\ \beta_{ij}\;\text{in}\;X_{j}∼\beta_{ij}X_{i}+pa_{j}, & X_{j}\notin pa_{i} \end{array}\right. \end{array}$$ (1)

*where X_j_ ∼ β_ij_ X_i_ + pa_j_ is the shorthand for the linear regression of X_j_ on X_i_ and pa_j_, and β_ij_ is the coefficient for X_i_ in the regression*.

Given the above theorem, we are able to estimate the regulatory effect of each miRNA on all mRNAs in a dataset, and use the mRNAs with top ranked causal effects as the targets of the corresponding miRNA. Note that because the available regulatory knowledge is very sparse, some edges in the causal structure may still remain undirected. Therefore we use the minimum absolute value as the estimation of the lower bound of the causal effect. We briefly summarise this procedure in Algorithm 2. For more details, please refer to the S6 File.

**Algorithm 2** Causal effects estimation

**Input**: Gene expression data **X**_*s* × *n*_, causal structure G.

**Output**: Causal effects matrix *C* where *C*(*i*, *j*) is the causal effect of miRNA_*i*_ on mRNA_*j*_.

 Initialize *C* as a zero matrix

 **for** All pairs of miRNA_*i*_ and mRNA_*j*_ **do**

  **for** All possible orientations of G **do**

   Calculate the causal effect with Theorem 1

  **end for**

  Let *C*(*i*, *j*) be the causal effect with lowest absolute value

 **end for**

 **return** *C*

### Evaluation methods

Evaluating miRNA target prediction methods is not an easy task. This is mainly because the current understanding of miRNA regulation mechanisms is still limited and experimentally validated target databases only contain information about frequently studied miRNAs. Therefore to evaluate the effectiveness of the CIDER framework, we use a number of different evaluation approaches described in the following:

1. We compare the predicted results to wet-lab validated miRNA target databases. Since CIDER needs access to regulatory knowledge, we reserve a part of the known regulatory relationships as the ground truth for evaluation. Specifically, when studying the performance of CIDER using TF-miRNA regulatory knowledge, we utilise the TF-miRNA interactions retrieved from TransmiR as the prior knowledge in constructing the causal structure and reserve the miRNA-mRNA interactions obtained from the miRNA target databases as the ground truth; when studying the effect of miRNA-mRNA regulatory knowledge, we utilise miRNA-mRNA interactions retrieved from TargetScan for causal structure construction and reserve the miRNA-mRNA interactions obtained from the experimentally validated miRNA target databases as the ground truth. In addition, if an interaction appears in the prior knowledge and the ground truth, we remove this entry from the knowledge and only use it for evaluation.
2. We compare the predicted targets to the results of miRNA transfection experiments. miRNA transfection is a technique that actively transfects a particular miRNA into cells, and by comparing the transfected expression profile to the controlled sample (same cell but without miRNA transfection), difference in mRNA expression level can be measured and mRNAs with top ranked logarithm fold change values can be considered as groundtruth miRNA targets [46].
3. We use gene pathway enrichment tools to analyse the functionality of predicted miRNA targets. It is often hypothesized that the predicted miRNA targets based on the expression profile should be closely related to the biological condition of the expression profiles. For example, the mRNAs targeted by miRNAs in the EMT dataset should be closely related to the epithelial to mesenchymal transition process. Therefore pathway functional analysis can be used to demonstrate the effectiveness of miRNA target prediction methods.

The above evaluations are used to demonstrate the effectiveness of CIDER for finding biologically relevant miRNA targets. To further demonstrate the performance CIDER when used with different amount of regulatory knowledge, we use the following simulation.

We simulate a gene regulatory networks and the corresponding gene expression profiles based on the linear structural equation model [47]. First we construct a directed graph where each node represents a miRNA or mRNA (including TF coding mRNA) in the regulatory network and the direction of an edge indicates that the parent node regulates the child node. Then we assign to each edge a weight *w*_*i*_ ($w_{i}∼U\left(\left[-1,-0.1\right]\cup \left[0.1,1\right]\right)$) which measures the amount of regulatory effect that the parent node has on the child node. Starting from the nodes without parents, we generate the expression value for each node following Gaussian distribution, with a non-Gaussian error terms added. Specifically the expression value of each gene is defined as follows:

$$\begin{array}{c} x_{i}=b_{i}+\sum_{j\in pa(x_{i})}w_{j}\cdot x_{j}+\epsilon_{i}, \end{array}$$ (2)

where *pa*(*x*_*i*_) denotes the parent nodes of *x*_*i*_, *w*_*j*_ ⋅ *x*_*j*_ is the regulatory effect of the *j*-th node has on the *i*-th one, *ϵ*_*i*_ represents the non-Gaussian error term of the *i*-th node, and *b*_*i*_ represents the interception term. To alleviate the effect of randomness in the simulated data, in total 50 networks (each of the network has approximately 1000 nodes) are generated and the average results from these 50 networks are reported. For each network we generate two sets of expression profiles, containing 250 and 500 samples, respectively.

To evaluate the performance on simulated datasets, we use F-Score (the harmonic mean of precision and recall) to measure the performance of all methods, which is formulated as follows:

$$\begin{array}{c} F=2\cdot \frac{precision\cdot recall}{precision+recall}. \end{array}$$

We use F-Score to compare CIDER with a variety of popular miRNA target prediction methods, including Pearson correlation [42], Lasso [48], Z-Score [49]. Pearson correlation calculates the correlation coefficients between pairs of miRNAs and mRNAs, and use the strength of the correlations to measure the regulatory effect. Lasso is a popular regression method which also measures linear correlation, but uses the L1-norm to overcome the sparseness of the high dimensional expression profiles. Z-Score is a specifically designed method to infer gene regulatory network using data from gene knock-out experiments. Since only observational data is used in our study, we use the lowest expression value of each gene among all sample as the value of knocked-out gene expression.

## Results and Discussions

### Transcriptional knowledge improves miRNA-mRNA target prediction

In this section, we investigate the effect of transcriptional TF-miRNA regulatory knowledge on miRNA target prediction. We first apply CIDER to analyse only the expression profiles, then we allow CIDER to access both TF-miRNA regulatory knowledge and the expression data and compare the performance of these two settings. For each miRNA, we consider the mRNAs with Top 50 and Top 100 ranked causal effects as its targets and compare them with those in the combination of three experimentally confirmed miRNA-mRNA interaction databases: Tarbase, miRWalk and miRTarbase.

**Fig 4:**
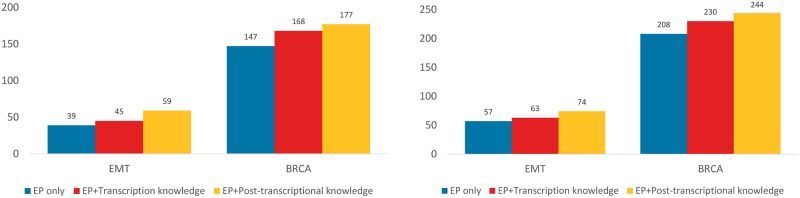
Number of experimentally validated miRNA targets (total number for all miRNAs) identified by CIDER when utilizing expression profiles (EP) only, EP + transcriptional regulatory knowledge, EP + post-transcriptional knowledge. (Left) Results for Top 100 predicted targets for each miRNA. (Right) Results for Top 150 predicted targets.

Although for both datasets only less than 20 of TF-miRNA interactions are integrated (the total number of possible edges is around 10^6^), it is evident to see the benefit of TF-miRNA knowledge for predicting miRNA targets. As shown in Fig 4, with the help of TF-miRNA regulation knowledge, CIDER predicts more validated miRNA targets than using expression profiles alone.

**Fig 5:**
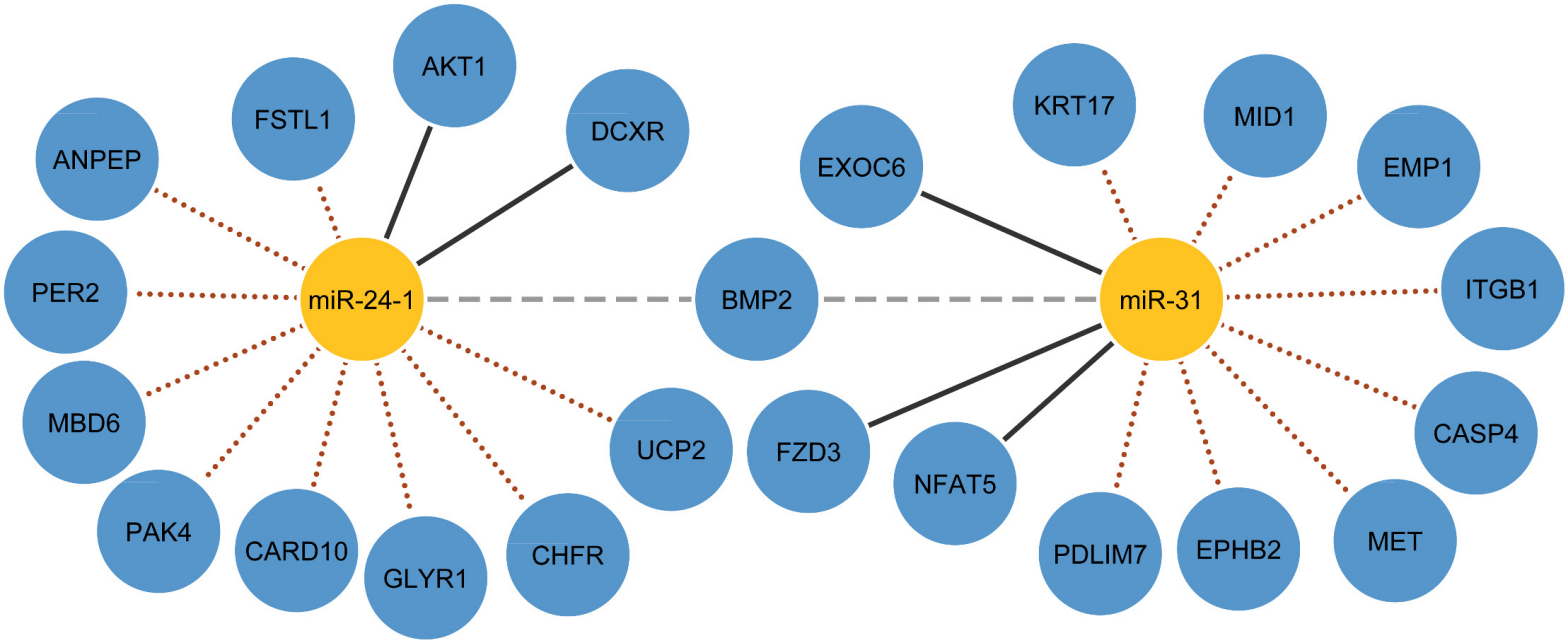
Comparison of miRNA targets identified by CIDER with and without TF-miRNA regulatory knowledge. Gray dashed lines indicate the TF-miRNA regulatory knowledge introduced from TransmiR. Black solid lines indicate miRNA-mRNA regulations found without knowledge. Brown dotted lines represent the additional miRNA-mRNA regulations found when TF-miRNA knowledge is utilised.

Fig 5 illustrates a comparison of the miRNA targets predicted by CIDER with and without TF-miRNA knowledge from both datasets. For example, without the TF-miRNA knowledge of BMP2→miR-31, only three predicted targets of miR-31 agrees with the experimentally validated database. However, when the TF-miRNA regulation between BMP2 is incorporated, CIDER not only successfully uncovers the up-regulation effect between BMP2 and miR-31, but also identifies 9 experimentally validated targets.

We conduct pathway enrichment analysis of the predicted target genes with the focus on KEGG pathways (adjusted p-value<0.05). To determine whether the top predicted miRNA targets are related to respective biological processes (EMT and BRCA), we select the top 5 predicted targets for each miRNA. As shown in Table 1, the KEGG pathways are highly associated with the relevant biological process. For instance, epithelial tight junctions are closely related to EMT process and focal adhesion is shown to be related to breast cancer in previous research [50].

**Table 1:** Top 10 enchriment KEGG pathways in the EMT and BRCA datasets. The p-values have been obtained through Hypergeometric analysis corrected by FDR method.

Datasets	Top 10 enrichment KEGG pathways	Adj-p-value
**EMT**	Epithelial tight junctions	5.95e-06
	Leukocyte transendothelial migration	1.82e-05
	Cell adhesion molecules	2.38e-04
	Arrhythmogenic right ventricular cardiomyopathy	3.23e-04
	Cell adhesion molecules	2.06e-03
	Melanogenesis	8.40e-03
	Regulation of actin cytoskeleton	9.74e-03
	Huntington’s disease	3.30e-02
	Pathways in cancer	1.07e-02
	Amoebiasis	1.07e-02
**BRCA**	Pancreatic secretion	1.20e-03
	Leukocyte transendothelial migration	1.83e-03
	Focal adhesion	2.32e-03
	Amoebiasis	4.94e-03
	Purine metabolism	5.19e-03
	Regulation of actin cytoskeleton	5.30e-03
	Salivary secretion	5.58e-03
	Adherens junction	5.58e-03
	Pathways in cancer	6.03e-03
	Tight junction	6.09e-03

### Post-transcriptional knowledge improves miRNA target prediction

In this section we show that post-transcriptional miRNA-mRNA knowledge improves the performance of CIDER. Similar to the previous section, we first apply CIDER to analyse the expression profiles alone, then compare it to the results obtained by allowing CIDER to access both the regulatory knowledge and the expression profiles.

Since we need to keep the experimentally validated target databases to evaluate the performance, miRNA-mRNA regulatory relationships predicted by TargetScan are used as the regulatory knowledge.

We depict the number of experimentally validated miRNA targets found by CIDER using expression profiles only and using both post-transcriptional regulatory knowledge and expression profiles in Fig 4. CIDER is able to successfully utilise the post-transcriptional knowledge and find significantly more validated targets than using expression profiles alone, despite that the regulatory knowledge in TargetScan contains false positives. The results not only demonstrate that CIDER is able to utilise post-transcriptional regulatory knowledge, but also indicate that CIDER can benefit from sequence-based prediction knowledge with false positives.

The reason behind the robustness of CIDER lies in the causal inference step. There the causal structure and expression profiles are analysed together to infer the amount of causal effects. If the false edges between miRNAs and mRNAs are not supported by the inference results, the noise introduced from false positive regulatory knowledge will be mitigated by the causal inference step.

When accessing all the experimentally validated miRNA target databases together with expression profiles, CIDER discovers more targets than accessing expression profiles alone. Since we use the databases as knowledge, other means are needed for evaluation. Therefore we compare the predicted targets for the EMT dataset to the transfection experiment on the MDA-MB-231 human cell line [41]. In this experiment, the gene expression level in the MDA-MB-231 samples transfected with hsa-miR-200a-3p/hsa-miR-200b-3p along with the expression level in those samples without hsa-miR-200a-3p and hsa-miR-200b-3p (control) were measured. (Please refer to S3 File for the detailed transfection experiment results). The differentially expressed genes from the controlled and transfected samples are used to validate the our computational predictions. Specifically, 345 and 533 genes are identified to be regulated by hsa-miR-200a-3p and hsa-miR-200b-3p, respectively.

**Fig 6:**
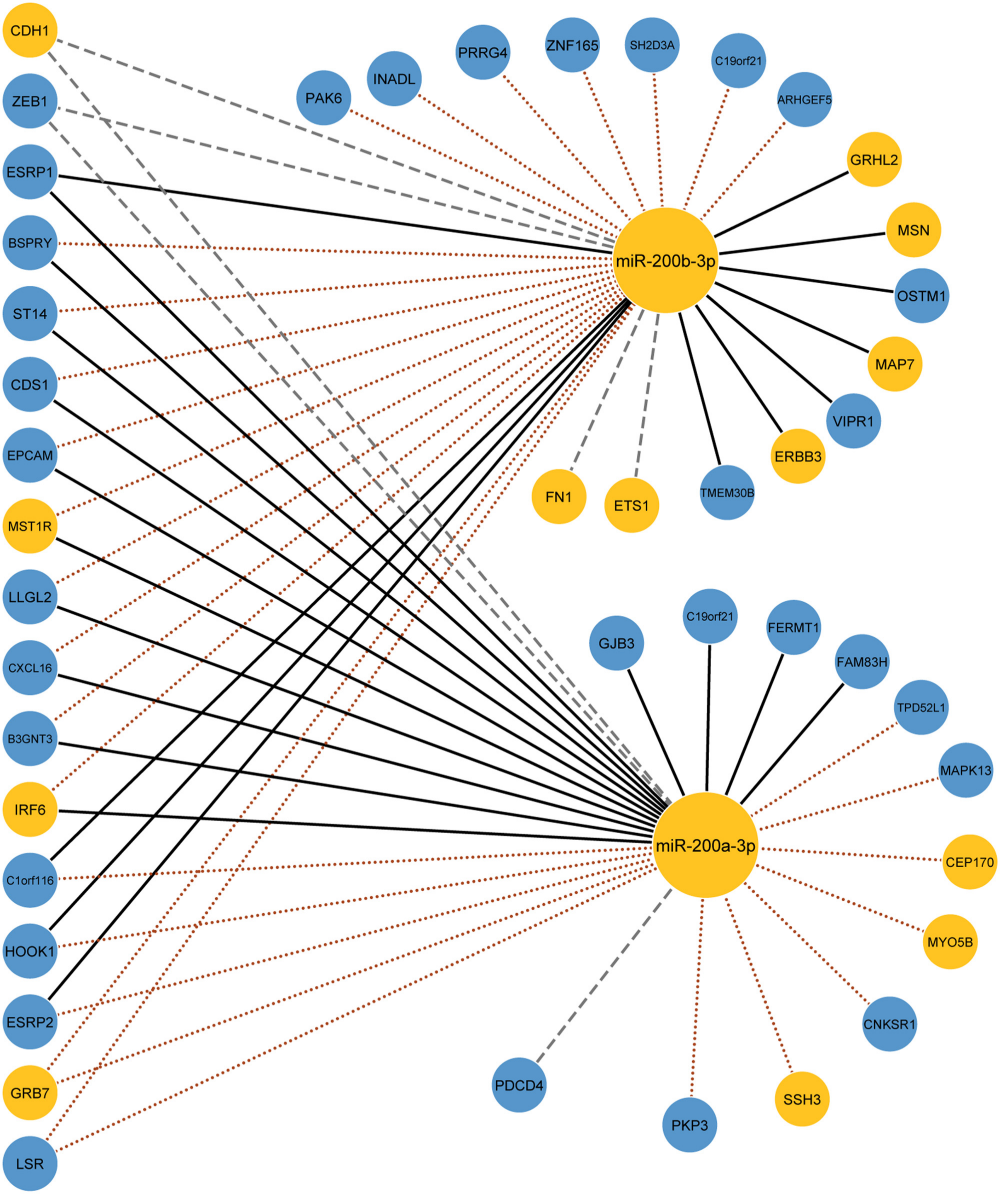
Comparison of validated regulatory relationships with/without regulatory knowledge on the EMT dataset. Black solid lines indicate validated interactions found with expression profiles; grey dashed lines indicate interactions provided by the regulatory knowledge; brown dotted lines indicate new interactions discovered by CIDER utilizing both expression profiles and regulatory knowledge, yellow shaded nodes are known oncogenes and oncomiRs according to [51].

The results demonstrate that with the help of post-transcriptional regulatory knowledge, CIDER identifies significantly more validated miRNA targets comparing to the miRNA targets predicted based only on expression profiles. Fig 6 shows that when equipped with the post-transcriptional miRNA-mRNA regulatory knowledge (brown dotted lines), CIDER is able to discover many novel miRNA-mRNA regulatory relationships that are missed by using expression data alone.

### More prior knowledge leads to better predictions

It is important to know that how the framework works with different amounts and types of regulatory knowledge. In this section we study the performance of CIDER when utilizing different amounts and types of knowledge. Since currently the wet-lab validated knowledge is very sparse, we generate the simulated networks and expression profiles as described in the Evaluation Methods section for our analysis.

**Fig 7:**
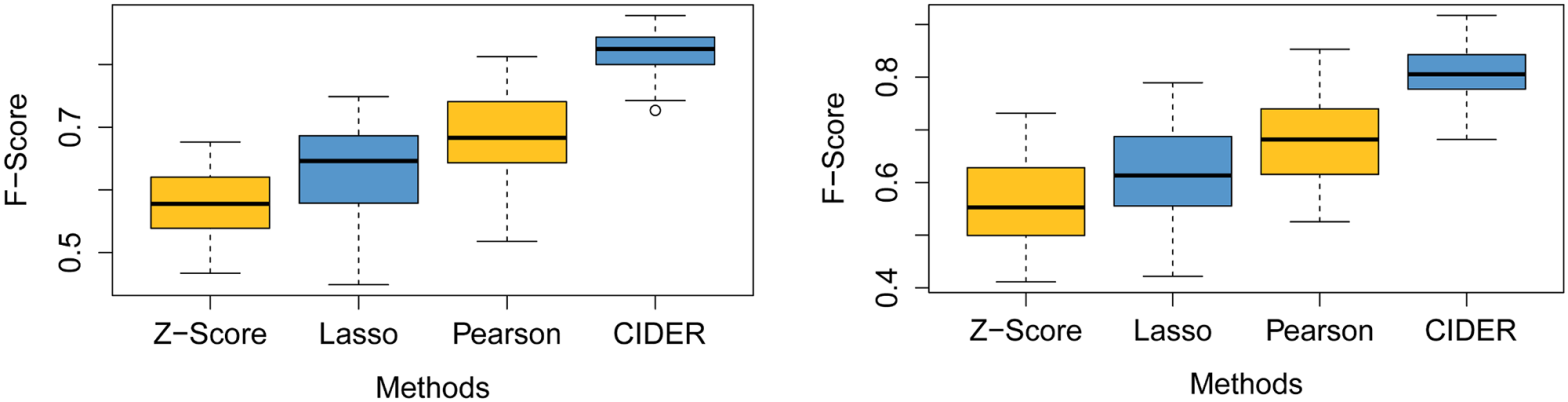
Comparing CIDER with Pearson, Lasso and Z-Score when only accessing expression profiles. Left: 250 samples; Right: 500 samples.

Even without knowledge, CIDER achieves comparable performance of state-of-the-art miRNA target prediction methods. As shown in Fig 7, when only utilizing the expression data, the performance of CIDER without prior knowledge is much better than Z-Score. Lasso and Pearson show similar performance regardless of the sparsity constraint added in Lasso. When comparing CIDER with Pearson and Lasso, even without using regulatory knowledge, CIDER shows slightly better performance than both methods because of CIDER utilised causation instead of correlation.

**Fig 8:**
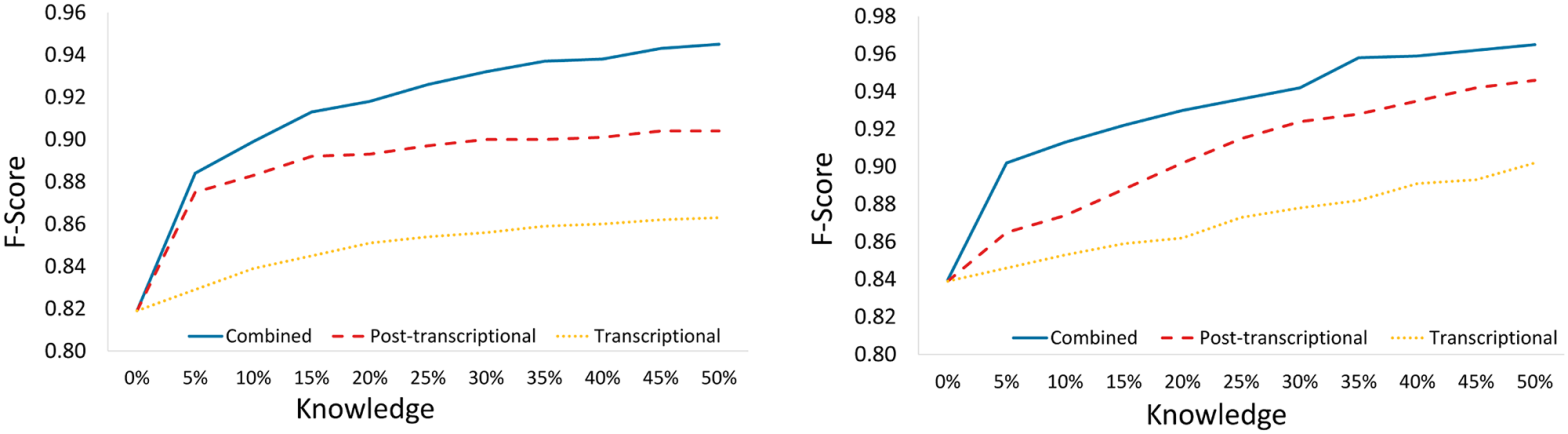
Performance of CIDER when utilizing different amounts and types of regulation knowledge. Sample size: 250 (left), 500 (right).

The performance of CIDER increases monotonically with the amount of knowledge. Combining post-transcriptional and transcriptional knowledge significantly boosts the performance of CIDER. To demonstrate this, we evaluate CIDER with three types of knowledge: miRNA-mRNA interactions, TF-miRNA interactions and the combination of these two. For each type of regulatory knowledge, starting from expression data only, we gradually increase the amount of knowledge available to CIDER from 0% to 50% (of the total amount of available knowledge of the type) by a 5% interval. As shown in Fig 8, both transcriptional and post-transcriptional knowledge separately improves the performance of CIDER significantly, and the combined knowledge leads to further improvement. For every type of regulatory knowledge, as the amount of utilised knowledge increases the performance of CIDER improves monotonically. With 50% of the combined knowledge, CIDER achieves very high accuracy.

In summary, CIDER is not only able to utilise either transcriptional or post-transcriptional regulatory knowledge to improve the performance of miRNA target prediction, but also able to utilise the combination of the two types of regulatory knowledge to further increase prediction accuracy. As the amount of regulatory knowledge increases, the performance of CIDER continuously improves. With this monotonic improvement, the miRNA target predicted by CIDER will become more accurate and reliable when our understanding of miRNA regulation improves and more knowledge is available for CIDER.

In return, CIDER can provide more precise guidance for selecting miRNA targets for wet-lab validation. Iteratively, as shown in Fig 1, CIDER will help to build a more and more complete gene regulation network.

### Methods utilizing sequence bindings information are not suitable for integrating experimentally validated knowledge

Methods designed to utilise sequence based predictions are not suitable for utilizing validated regulatory knowledge. In this section we compare CIDER with ProMISe [30], a recently proposed method designed to utilise sequence binding information and expression profiles.

We compare two algorithms on the EMT and BRCA datasets. Both algorithms have access to the expression profiles, and exactly the same amount of regulatory knowledge, which contains the sequence binding interactions predicted by TargetScan, experimentally validated post-transcriptional knowledge in miRWalk and miRTarbase. Specifically, ProMISe uses the knowledge as sequence binding information, while CIDER uses it to initialise constant edges.

**Fig 9:**
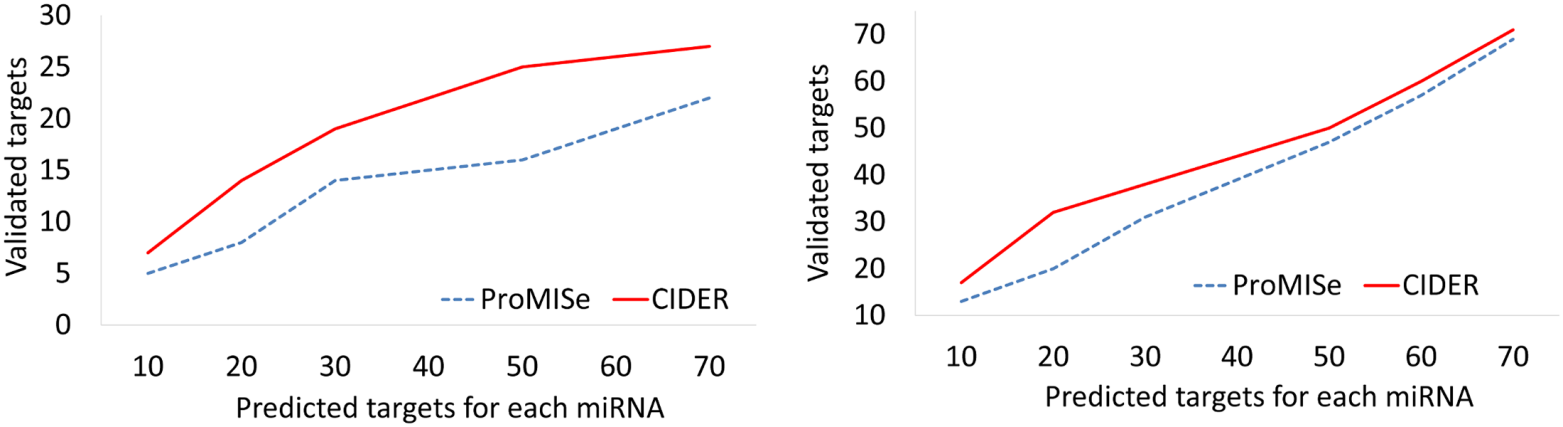
Performance comparison of CIDER and ProMISe when utilizing post-transcriptional regulation knowledge. Left: EMT dataset, right: 500 BRCA dataset.

As can be seen in Fig 9, regardless of what threshold is selected for the miRNA targets, CIDER discovers more validated target than ProMISe. This results indicate that the top miRNA targets predicted by CIDER are consistently better than the ones predicted by ProMISe.

The reason is that instead of considering all possible miRNAs and mRNA pairs, ProMISe (and other similar algorithms) uses sequencing information to constrain their search space. In other words, a miRNA-mRNA interaction would not be considered unless the pair is included in the knowledge. Therefore when utilizing sequencing information, these algorithms will be misled by the false negatives; when utilizing experimentally validated knowledge, they will only predict interactions that are already included in the knowledge.

### Putative miRNA targets

In this section, we report the high-confidence miRNA targets predicted by CIDER in the EMT and BRCA datasets for biological researchers to explore. These predictions utilise expression profiles with both transcriptional and post-transcriptional regulatory knowledge. As we have shown in the previous section, CIDER performs better when utilizing the combined knowledge than using either type of regulatory knowledge separately. Therefore, we expect that the miRNA targets predicted by CIDER utilizing TF-miRNA interactions from TransmiR and miRNA-mRNA knowledge from Tarbase, miRTarbase, miRWalk, should provide valuable putative candidates for further biological wet-lab evaluation. To utilise sequence binding information to increase the confidence of the predicted targets, we intersect our discovery with miRNA target prediction from TargetScan.

**Fig 10:**
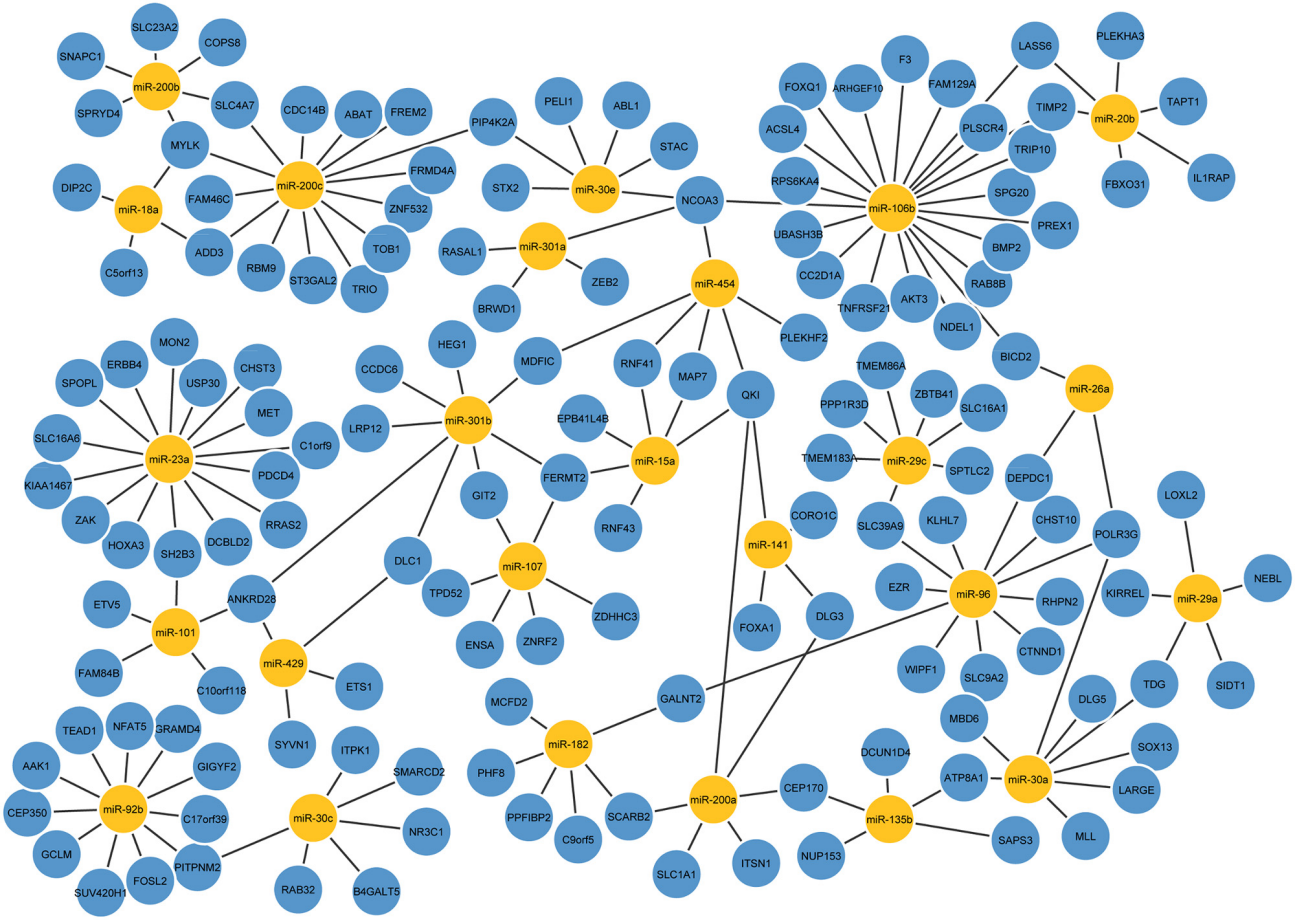
High confidence miRNA targets predicted by CIDER utilizing expression profiles, transcriptional and post-transcriptional knowledge. Only part of the interactions are shown for clarity of illustration, please refer to S5 File for the full results.

These high-confidence predicted miRNA targets are presented in Fig 10, and we hope that a significant number of them will be validated by experiments in the future.

## Conclusion

The future of biology is neither based on wet-lab experiments nor computational predictions alone, but on their combination. The progress of wet-lab experiments would be hampered without the help of quality computational predictions, and the power of computational methods would be limited if accumulated biological knowledge were not integrated with the modeling process.

In this article, we present the CIDER framework that seamlessly integrates biological knowledge with high-throughput expression profiles for miRNA target prediction. We use a causal Bayesian network based method to explicitly exploit experimentally validated gene regulatory knowledge to improve the prediction of miRNA-mRNA interactions. Our results demonstrate that when utilizing transcriptional or post-transcriptional knowledge, CIDER discovers significantly more validated miRNA targets than using expression profile alone. Furthermore, when the amount of available regulatory knowledge increases, the performance of CIDER increases monotonically.

With the capability to improve prediction accuracy with the increment of gene regulatory knowledge, our causal discovery framework can serve as a promising tool for uncovering new biological insights using ever increasing regulatory knowledge and new high-throughput data.

## Supporting Information

S1 File Differential expression profiles of miRNAs and mRNAs for the EMT and BRCA datasets. The p-values are adjusted by Benjamini-Hochberg (BH) method.
(XLSX)

S2 File R source code for the proposed CIDER framework. (ZIP)

S3 File Experimentally validated miRNA-mRNA regulatory knowledge. This file includes the miRNA-mRNA regulatory knowledge obtained from the following databases: TarBase, miRecords, miRWalk and miRTarBase.
(XLSX)

S4 File miRNA transfection result on MDA-MB-231 samples. This file includes the transfection results for hsa-miR-200a and hsa-miR-200b, and control sample.
(XLS)

S5 File High-confidence miRNA targets predicted by CIDER. This file includes the miRNA targets predicted by CIDER when utilizing post-transcriptional and transcriptional regulatory knowledge and expression profiles, these interactions are also predicted by TargetScan v7.0.
(XLSX)

S6 File Detailed descriptions of Algorithm 1 and Algorithm 2, and additional validation results. (PDF)
