# Supplementary material for: Predicting miRNA Targets by Integrating Gene Regulatory Knowledge with Expression Profiles
Source: PLoS One. 2016 Apr 11;11(4):e0152860. doi: 10.1371/journal.pone.0152860 (PMC4827848; doi:10.1371/journal.pone.0152860)
Supplement: S6 File — (PDF) [file pone.0152860.s006.pdf]

Predicting miRNA targets by integrating gene regulatory knowledge with expression profiles: supplementary material

In this supplementary material, we provide the algorithm details for the main paper. We also provide additional validation results.

1 Algorithm 1

We formally summarize the procedure of causal structure construction. Suppose that we are interested in the regulatory relationships among p miRNAs and q mRNAs represented as $\mathbf{X} = \{X_1, \ldots, X_p, X_{p+1}, \ldots, X_{p+q}\}$, where $X_1, \ldots, X_p$ denote the miRNAs and $X_{p+1}, \ldots, X_{p+q}$ denote the mRNAs (including TF coding mRNAs). Given the expression profile data $\mathbf{X}_{s\times n}$ of s samples of $n = p + q$ miRNAs and mRNAs, the prior knowledge matrix $\mathbf{M}_{n\times n}$ where an entry with $m_{i,j} = 1$ indicates regulatory relationship between the ith to jth gene in the prior knowledge, let ci_test represent the conditional independence test procedure in the PC algorithm. $ci\_test(i, j) = 0$ if the ith and jth variables are dependent given any conditional set, and $ci\_test(i, j) = 1$ if they are independent given a conditional set $\mathbf{S}$, we describe the details for constructing the causal structure in Algorithm 1.

2 Algorithm 2

We summarize the details of Algorithm 2 in this section. Given the expression profile matrix $\mathbf{X}$, and the causal structure constructed by Algorithm 1, our goal is to estimate a matrix $\mathbf{C}$ where each entry $C(i, j)$ represents the amount of causal effect that miRNA$_i$ has on mRNA$_j$.

3 Additional results when utilizing transcriptional knowledge

To demonstrate the effectiveness of CIDER when utilizing transcriptional knowledge, in Fig. 1 we show more miRNA targets predicted with CIDER using expression profiles and TransmiR.

Algorithm 1 Construct the causal structure $\mathcal{G}$

Require: Gene expression profile data $\mathbf{X}_{s\times n}$, prior knowledge matrix $\mathbf{M}_{n\times n}$.
Ensure: Constructed causal graph $\mathcal{G}$
  $\mathcal{G} \leftarrow$ fully connected graph with n vertices
  //Constructing the graph with prior knowledge
  for All $i, j (i \neq j) \in \{1, \ldots, n\}$ **do**
    $cond\_set(i, j) \leftarrow$ NULL
    if $m_{i,j} \neq 1$ **AND** $ci\_test(i, j) = 1$ **then**
      Remove the edge between X_i and X_j
      //save the conditional set **S** returned by $ci\_test(i, j)$
      $cond\_set(i, j) \leftarrow \mathbf{S}$
    else if $m_{i,j} = 1$ **then**
      Orient $X_i - X_j$ into $X_i \rightarrow X_j$
    end if
  end for
  for All pairs of nonadjacent X_i and X_k with common neighbor X_j **do**
    //Determining the v-structure
    if $X_j \notin cond\_set(i, k)$ **AND** $X_i - X_j - X_k$ **then**
      Orient $X_i - X_j - X_k$ into $X_i \rightarrow X_j \leftarrow X_k$
    end if
  end for
  //Repeatedly apply the following rules to orient as many edges as possible
  if $X_j - X_k$ **AND** $X_i \rightarrow X_j$ **AND** $cond\_set(i, k) \neq$ NULL **then**
    Orient $X_j - X_k$ as $X_j \rightarrow X_k$
  end if
  if $X_i \rightarrow X_k \rightarrow X_j$ **AND** $X_i - X_j$ **then**
    Orient $X_i - X_j$ as $X_i \rightarrow X_j$
  end if
  if $X_i - X_k$**AND**$X_i - X_k \rightarrow X_j$ **AND** $X_i - X_l \rightarrow X_j$ **AND** $cond\_set(k, l) \neq$ NULL **then**
    Orient $X_i - X_j$ as $X_i \rightarrow X_j$
  end if
  if $X_i - X_k \rightarrow X_l \rightarrow X_j$ **AND** $X_i - X_j$ **AND** $cond\_set(k, j) \neq$ NULL **then**
    Orient $X_i - X_j$ as $X_i \rightarrow X_j$
  end if
  return $\mathcal{G}$

4 Additional experiments using post-transcriptional knowledge

In Table 1 we show the additional results of CIDER utilizing post-transcriptional knowledge and expression profiles. Particularly, we utilize the regulatory knowledge from the miRNA target predicted by miRANDA [1], and the expression profiles described in the main article. To validate the results, we used the same

Algorithm 2 Estimate the causal effects between miRNA$_i$ and mRNA$_j$

Require: Gene expression profile data $\mathbf{X}_{s\times n}$, causal structure $\mathcal{G}$.
Ensure: Causal effects matrix C where $C(i,j)$ is the causal effect of miRNA$_i$ on mRNA$_j$.

```
C ← n × n zero matrix
Determine all possible causal DAGs G_1, ..., G_m by iterating directions over undirected edges in 𝒢
for i = 1 to p do
  for j = p + 1 to p + q do
    for t = 1 to m do
      θ_ijt = β_ij|pa_j(G_t)
    end for
    C(i, j) = min_{t∈1,...,m} |θ_ijt|
  end for
end for
return C
```

combined experimentally validated databases as in the main article.

Similar to TargetScan, miRANDA also utilizes sequence binding information to predicted miRNA/mRNA binding sites, and use the mRNAs with corresponding miRNA binding sites as the predicted miRNA target.

The results show that CIDER is able to utilize miRANDA to improve prediction performance, despite that the knowledge in miRNADA is also prone to false positives.

Table 1: Number of validated miRNA target discovered by CIDER when utilized expression profiles (EP) only, and utilizing EP with miRANDA (EP+miRANDA). Best results for respective datasets are bolded.

	Top100	Top150	Top 200	Top 250	Top 300
EMT(EP)	26	39	57	72	85
EMT(EP+miRANDA)	**27**	**42**	**60**	**78**	**90**
BRCA(EP)	106	147	208	261	313
BRCA(EP+miRANDA)	**109**	**166**	**223**	**286**	**330**

5 Pathway analysis for predicted miRNA targets

We show the results of pathway analysis for the miRNA targets predicted by CIDER when utilizing all three types of knowledge in Table 2.

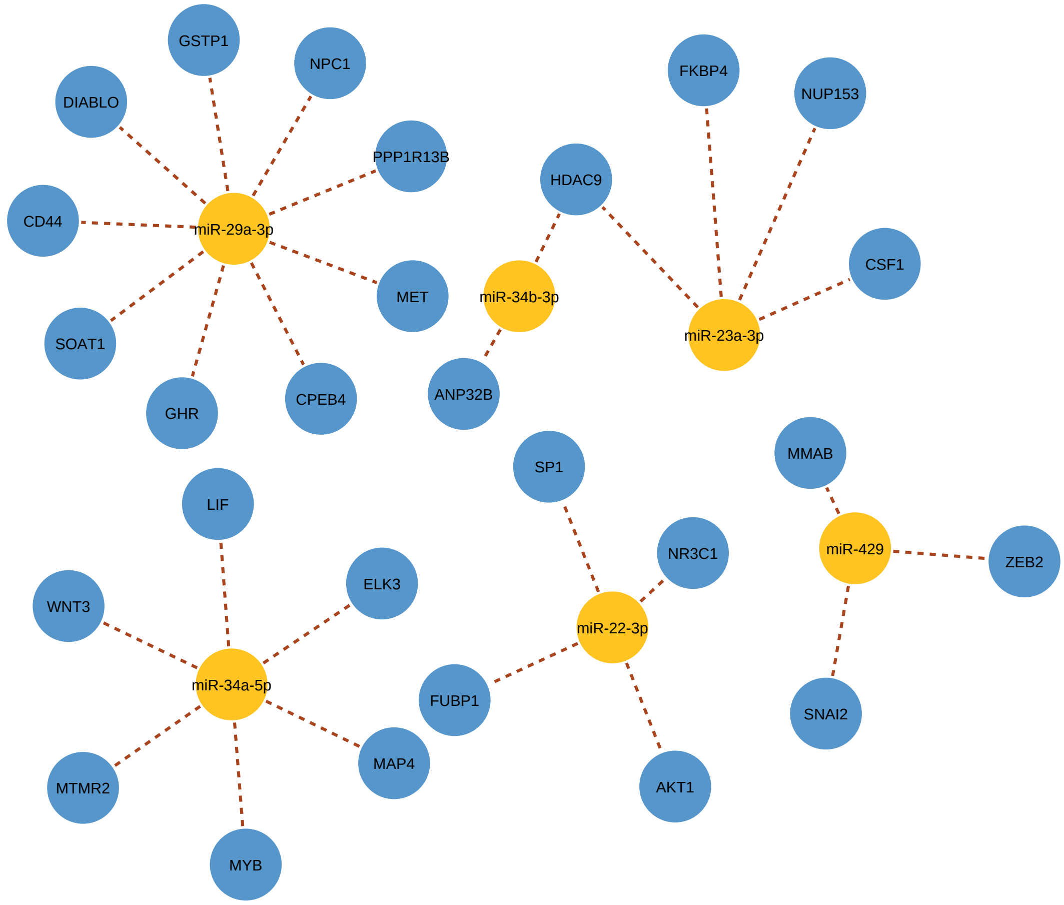

Figure 1: Comparison of miRNA targets identified by CIDER with and without TF-miRNA regulatory knowledge. Gray dashed lines indicate the TF-miRNA regulatory knowledge introduced from TransmiR. Black solid lines indicate miRNA-mRNA regulations found without knowledge. Brown dotted lines represent the additional miRNA-mRNA regulations found when TF-miRNA knowledge is utilized.

References

[1] Betel D, Wilson M, Gabow A, Marks DS, Sander C. The microRNA.org resource: targets and expression. Nucleic Acids Res. 2008;36:D149–53.

Table 2: **Top 15 enchriment KEGG pathways for the predicted miRNA targets.** The p-values have been obtained through Hypergeometric analysis corrected by FDR method.

**Top 10 enrichment KEGG pathways**	**Adj-p-value**
Focal adhesion	1.19e-04
Pathways in cancer	1.45e-03
Melanoma	1.81e-03
Renal cell carcinoma	1.81e-03
Amoebiasis	2.03e-03
ECM-receptor interaction	2.30e-03
Mucin type O-Glycan biosynthesis	2.74e-03
Cytokine-cytokine receptor interaction	2.79e-03
Neurotrophin signaling pathway	3.37e-03
Endocytosis	3.58e-03
MAPK signaling pathway	3.90e-03
Small cell lung cancer n	4.70e-03
Regulation of actin cytoskeleton	4.93e-03
Lysine degradation	6.82e-03
Adherens junction	1.68e-02
